# Comparison of Early Postoperative Recovery and Radiologic Outcomes Between Microscopic and Unilateral Biportal Endoscopic Posterior Cervical Foraminotomy for Cervical Radiculopathy

**DOI:** 10.3390/jcm15124589

**Published:** 2026-06-12

**Authors:** Sang Youp Han, Sang Hyub Lee, Jae Won Jang, Choon Keun Park, Dong Geun Lee

**Affiliations:** 1 Department of Neurosurgery, Spine Center, The Leon Wiltse Memorial Hospital, Suwon 16542, Republic of Korea; 2 Department of Neurosurgery, Seoul Central Hospital, 101, Daehak-ro, Jongno-gu, Seoul 03080, Republic of Korea

**Keywords:** posterior cervical foraminotomy, unilateral biportal endoscopy, endoscopic spine surgery, cervical radiculopathy, minimally invasive surgical procedures

## Abstract

**Objective**: This study aimed to compare the clinical and radiological outcomes between microscopic and unilateral biportal endoscopic (UBE) posterior cervical foraminotomy (PCF). **Methods**: This study included 73 patients who underwent microscopic PCF (*n* = 40) or UBE PCF (*n* = 33) for single-level cervical foraminal disc herniation or stenosis between January 2018 and December 2021. Clinical outcomes were measured using the Visual Analog Scale (VAS) and Neck Disability Index (NDI). Radiologic outcomes were evaluated with cervical range of motion (ROM) using computed tomography and flexion-extension dynamic radiography. **Results**: The mean follow-up period for microscopic and UBE PCF was 33.0 ± 7.6 months and 29.9 ± 5.9 months, respectively. The postoperative neck VAS until postoperative 2 weeks was significantly lower in the UBE PCF group than in the microscopic PCF group (*p* < 0.05). The estimated blood loss and operative time were significantly lower in the UBE PCF group than in the microscopic PCF group, while the length of hospital stay was numerically shorter but did not reach statistical significance. The two groups had no significant difference in the NDI on the preoperative and postoperative 3 months. The recurrence occurred in 1 patient (2.5%) of the microscopic PCF group and 1 patient (3%) of the UBE PCF group. The revision surgery was performed in 2 patients (5%) of the microscopic PCF group and in 1 patient of the UBE PCF group. There were no significant differences in motion and instability between the two groups. **Conclusions**: Both microscopic and UBE PCF are effective and safe procedures for treating cervical radiculopathy due to cervical foraminal disc herniation or stenosis. The UBE approach may provide advantages mainly in early postoperative recovery, including lower early postoperative neck pain, while long-term clinical and radiologic outcomes appear comparable to those of microscopic PCF.

## 1. Introduction

Cervical radiculopathy is a common condition caused by foraminal disc herniation or degenerative foraminal stenosis. Although anterior cervical discectomy and fusion (ACDF) has been widely used as a standard surgical treatment, it inevitably sacrifices motion at the treated segment and may increase mechanical stress at adjacent levels. In contrast, posterior cervical foraminotomy (PCF) allows direct decompression of the affected nerve root while preserving segmental motion, and has therefore been increasingly performed for selected patients with unilateral cervical radiculopathy caused by foraminal disc herniation or foraminal stenosis [1,2,3,4,5].

The posterior approach also has the advantage of avoiding injury to the tracheoesophageal complex and preventing motion limitation related to spinal fusion [3,6,7,8,9,10]. However, conventional open microscopic PCF requires posterior muscle dissection and partial removal of the facet joint, which may contribute to postoperative neck and shoulder pain, muscle injury, bleeding from the venous plexus, and concerns regarding postoperative instability [7,11,12,13]. In particular, venous bleeding may interfere with the operative field and make delicate neural decompression more difficult.

Minimally invasive endoscopic approaches have therefore been introduced to reduce tissue disruption while maintaining adequate neural decompression. Unilateral biportal endoscopic (UBE) PCF has emerged as an alternative technique for addressing these limitations. UBE PCF provides a wide and clear operative field through continuous irrigation and allows the use of familiar surgical instruments through a separate working portal. These technical features may reduce venous bleeding, minimize injury to the posterior neck muscles and ligaments, and facilitate early postoperative recovery. More broadly, minimally invasive surgical procedures may reduce surgical trauma by minimizing soft tissue injury, bleeding, and postoperative inflammatory responses, which may contribute to faster postoperative recovery [14].

However, although several studies have reported favourable outcomes after endoscopic PCF, comparative evidence directly evaluating microscopic PCF and UBE PCF remains limited, particularly with regard to both early clinical recovery and radiologic changes after surgery. Therefore, this study aimed to compare the clinical and radiological outcomes of UBE PCF with those of conventional microscopic PCF in patients with cervical radiculopathy caused by single-level foraminal disc herniation or foraminal stenosis.

## 2. Materials and Methods

This study was approved by the Institutional Review Board of Wiltse memorial hospital (IRB No. 2022-W14). The requirement for informed consent was waived by the IRB due to the retrospective nature of the study.

### 2.1. Materials

We retrospectively reviewed the patients who underwent surgery for cervical radiculopathy at our hospital between January 2018 and December 2021. After applying the inclusion and exclusion criteria, 73 patients were included in the final analysis, including 40 patients in the microscopic PCF group and 33 patients in the UBE PCF group. Consecutive patients who met the inclusion and exclusion criteria during the study period were included. Because UBE PCF was introduced later at our institution, the choice of surgical technique was mainly determined by the treatment period and surgeon preference rather than by randomized allocation. No specific selection criteria were intentionally applied to favour either microscopic PCF or UBE PCF, and the operative indication, level, and side were determined based on the correlation between clinical symptoms, neurologic examination, and imaging findings. We included the patients with cervical radiculopathy caused by single level cervical disc herniations or foraminal stenosis. We excluded the patients with segmental instability, myelopathy, operative history, fracture in the cervical region, infection, ossification of the posterior longitudinal ligament, or multilevel pathology. Surgical indications included unilateral cervical radiculopathy with concordant radiologic findings on MRI or CT, caused by single-level foraminal disc herniation or foraminal stenosis, and persistent symptoms despite conservative treatment or the presence of neurologic deficits.

We investigated the demographic characteristics, operative level, classification of pathologies, operative time, length of hospital stay, and estimated blood loss. Data analysis was performed using SPSS ver 28.0 (SPSS Inc., Chicago, IL, USA).

### 2.2. Clinical Outcomes

Clinical outcomes were measured using the Visual Analog Scale (VAS) for neck and arm pain, and Neck Disability Index (NDI). Neck and arm VAS scores were measured preoperatively and at postoperative day (POD) 0, POD 3, POD 14, and POD 3 months. The NDI was measured preoperatively, POD 2 weeks and 3 months. All clinical outcome measures were collected using the same assessment schedule in both groups during hospitalization and outpatient follow-up. In addition, change-score analyses were performed for NDI to account for baseline differences, and ΔNDI was calculated as postoperative NDI minus preoperative NDI. The same standardized discharge criteria were applied to both groups, including adequate pain control with oral medication, stable neurological status, absence of wound-related complications, and independent ambulation. Estimated blood loss was calculated as the volume collected in the suction canister after subtracting the amount of irrigation fluid used. In the UBE PCF group, because continuous irrigation was used, the irrigation inflow volume was recorded and subtracted from the total suction volume to estimate blood loss. Gauze assessment was also considered, and the same estimation protocol was applied to both groups.

### 2.3. Radiologic Outcomes

Radiologic outcomes were evaluated with preoperative and postoperative radiological images, including plain radiography, computed tomography (CT), and magnetic resonance imaging (MRI). The C2–7 cervical lordosis, segmental Cobb angle, and disc height change were measured on the plain radiograph. Postoperative flexion-extension dynamic radiographs were routinely obtained at the final follow-up in all patients, and the same radiographic follow-up protocol was applied to both groups. Segmental instability was defined as more than 3.5 mm of translation or more than 11° of angular motion at the operated segment on flexion-extension radiographs. Cervical lordosis was measured as the C2–7 Cobb angle on neutral lateral radiographs. The segmental Cobb angle was measured at the operated level using the adjacent vertebral endplates. Disc height was measured at the index level on lateral radiographs. Changes in radiologic parameters were calculated as postoperative values minus preoperative values. These radiologic parameters were selected as commonly used surrogate markers to assess sagittal alignment, segmental stability, and potential postoperative instability following posterior cervical foraminotomy. Postoperative MRI was routinely obtained one day after surgery to evaluate complications such as hematoma or remnant disc material. All radiologic measurements were performed by a single experienced spine surgeon using standardized measurement techniques to ensure consistency. Although formal interobserver or intraobserver reliability analysis was not performed, these radiologic parameters have been reported to show acceptable reproducibility in previous studies [15,16].

### 2.4. Operation Procedures

#### 2.4.1. Microscopic PCF

Microscopic PCF was performed with a 3 cm longitudinal skin incision. After muscle dissection, laminotomy and foraminotomy were performed using a high-speed drill and punches not to resect more than half of the facet joint. After confirming adequate nerve root decompression with a fine dissector, we completed the operation. The extent of laminectomy and facetectomy differ according to the location of the disc material and foraminal stenosis. The epidural venous plexus was coagulated using bipolar cautery, beginning medially and extending along the nerve root sleeve [16]. A pediculectomy was performed at 2–3 mm, and compressive disc fragments and osteophytes were then removed using pituitary forceps or curettage without excessive retraction of the nerve root. Hemostasis was most achieved using thrombin-mixed Cutanplast (Mascia Brunelli S.p.A., Milan, Italy).

#### 2.4.2. Biportal Endoscopic PCF

**Figure 1:**
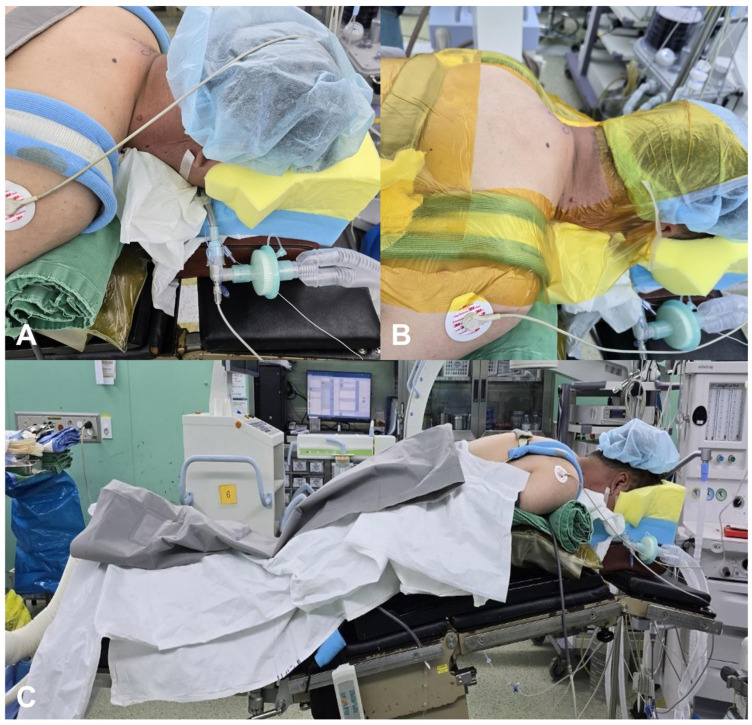
(**A**) The eyeball must be protected by a gel facial pad; (**B**) Neck and bilateral shoulders must be fixed with plaster or clavicula bandage, without headrest; (**C**) Surgical position for PCF. The upper back must be slanted down, because of good venous return.

**Figure 2:**
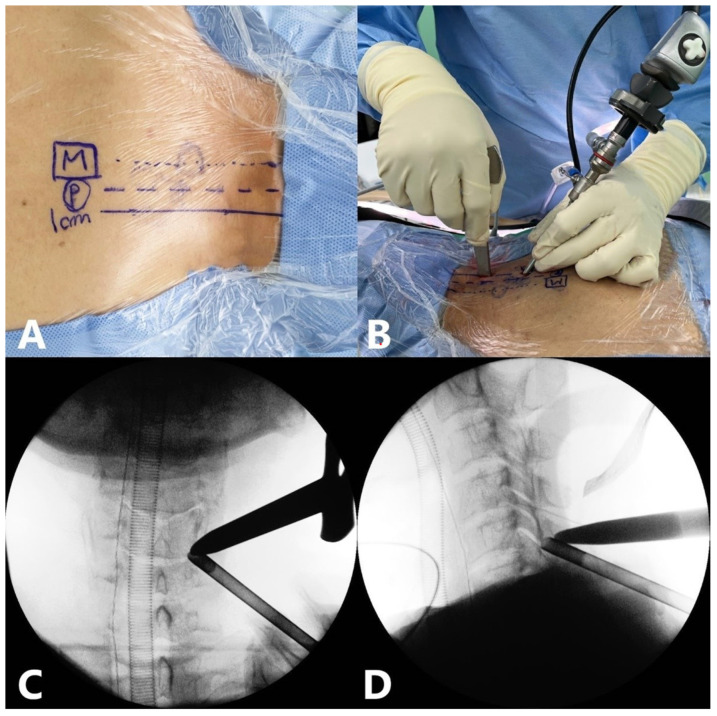
Surgical procedures of UBE posterior cervical foraminotomy. (**A**) Skin incision sites for the 2 portals on the C-arm image and on the patient’s neck (dotted line: midline, dashed line: pedicle lateral margin, solid line: 1 cm distance of pedicle). (**B**) View of surgical scene. (**C**) anteroposterior (AP) view of the C-arm. (**D**) Lateral view of the C-arm.

**Figure 3:**
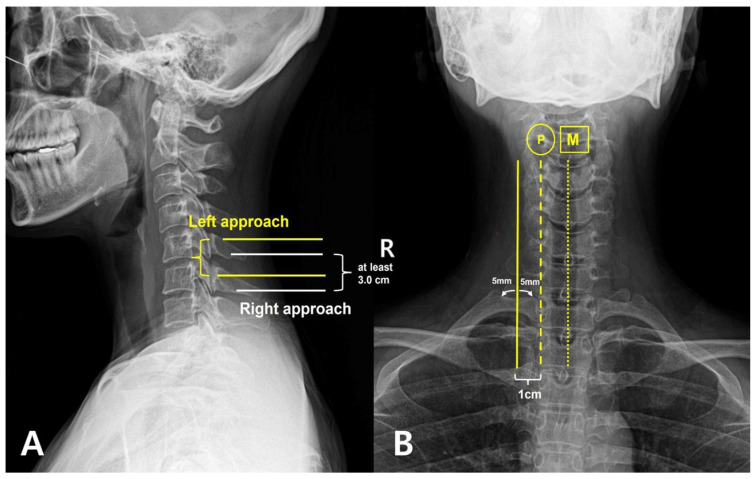
(**A**) The skin incision points of PCF are marked on the upper and lower pedicles around the target level. In the case of the right approach, it is convenient to lower the vertebral body in the caudal direction. It is easier to operate with at least 3 cm of space between the ports. (**B**) In anteroposterior view, midline, pedicle lateral margin and pedicle lateral 1 cm are indicated, and the location of the portal is selected according to the lesion. If the location of the lesion is paracentral, it is convenient to start at pedicle 1 cm lateral, and if the lesion is on the distal foramen side, it is convenient to enter the medial about 0.5 cm.

The operation was performed in a prone position under general anesthesia with the head slightly flexed on a radiolucent frame. After placing the waterproof drape, the surgical level was checked using C-arm fluoroscopy in the lateral view (Figure 1). Biportal endoscopic PCF cases utilized the usual biportal endoscopic systems with a 4.0 mm diameter, zero-degree optic view angle endoscope. The procedure was performed using the pedicles above and below the target level as anatomical landmarks (Figure 2). If the pathology is the right approach, operating from slightly below is convenient. The scope and working port were each placed at least 2.5 cm to 3.0 cm apart to avoid cross-interference. In the anteroposterior view, starting point is about 0.5–1 cm outside point V where the lamina meets the lateral mass considering each patient’s neck depth (Figure 3).

**Figure 4:**
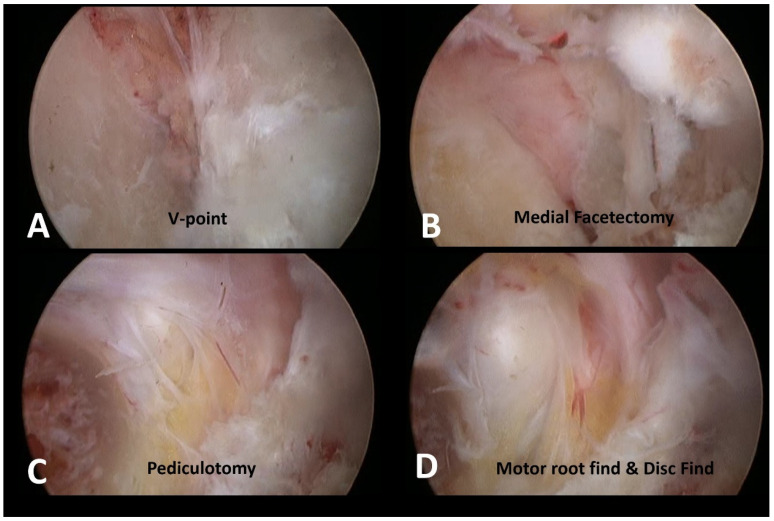
Surgical procedure for UBE posterior cervical foraminotomy. (**A**) Identification of the V-point, where the lamina meets the lateral mass. The yellow arrow indicates the V-point. (**B**) Medial facetectomy. (**C**) Pediculotomy. The yellow line indicates the pedicle margin. (**D**) Identification of the motor root and disc fragment.

**Figure 5:**
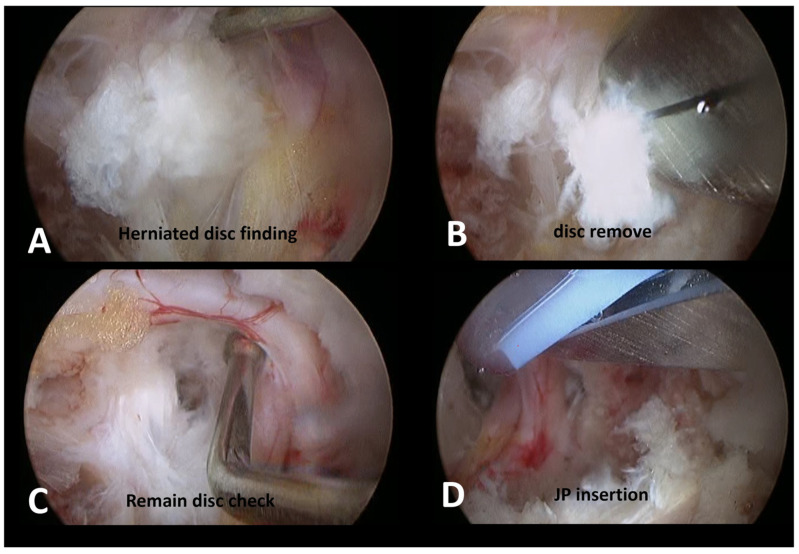
UBE posterior cervical foraminotomy procedure. (**A**) Herniated disc visualized with a hook. (**B**) Disc fragment removal. (**C**) Confirmation of remnant disc. (**D**) JP drain insertion.

Approximately 1 cm of the working portal and 0.5 cm of the scope portal are sufficient for incision. It is important to note that the posterior neck has many muscles and layers of fascia; deep incisions should be made, and sufficient incisions should be made to produce water outflow. Muscle splitting was performed using a sequential dilator with minimal injury to the 3 layers of muscles of the cervical area. Subsequently, a T-handle was used to achieve sufficient detachment, and use of a retractor makes outflow easier because the posterior cervical region is deep. The skin incision is recommended under the fluoroscopic image guide. The lamina, which serves as the fixed point for the scope, and the V-point, which is the medial junction of the facet joints, was used as a surgical anatomical landmark to identify the anatomy. Drilling can be initiated at checking point V. The upper lamina and half facet are drilled until the tip of the lower facet is visible. The lower facet was then drilled, taking care not to violate more than half of the facet joint. The cortical bone was drilled very thinly taking care not to damage the neural structure. Subsequently, identifying the cancellous bone of the inferior pedicle is important while drilling the pedicle, the cord and root contours clearly appear (Figure 4). The ruptured disc fragment was mainly removed in the axillary portion of root which consists of each motor and sensory. Bleeding was controlled using bipolar radiofrequency coagulation under continuous saline irrigation. After bleeding control, the Jackson-Pratt (JP) drain was inserted, and the operation was completed (Figure 5).

### 2.5. Statistical Analysis

Statistical analysis was performed using SPSS ver 28.0 (SPSS Inc., Chicago, IL, USA). A *p*-value < 0.05 was considered statistically significant. Continuous variables were presented with mean ± standard deviation. Continuous variables were compared between microscopic PCF and UBE PCF using *t*-test or Wilcoxon rank sum test according to result of normal distribution test. Categorical variables were presented with number of counts with percentages. Categorical variables were compared using chi-square test. Only patients with complete clinical data at each evaluated time point were included in the analysis, and no imputation for missing data was performed.

## 3. Results

A total of 73 patients were included in this study, with 40 undergoing conventional microscopic PCF and 33 undergoing UBE PCF. Microscopic PCF procedures were performed between January 2018 and December 2019, whereas UBE PCF procedures were performed between May 2019 and December 2021. Although the mean follow-up durations differed slightly between the two groups (33.0 ± 7.6 vs. 29.9 ± 5.9 months, *p* = 0.061), all patients were followed for at least 24 months, and recurrence, revision surgery, postoperative complications, and radiologic outcomes were assessed during this follow-up period. The mean age of microscopic PCF (56.6 ± 9.7) was higher than UBE PCF (56.1 ± 10.0) (*p* = 0.863). The sex distribution was also comparable between the two groups, with 25 males and 15 females in the microscopic PCF group and 22 males and 11 females in the UBE PCF group. The most common surgical level was C6/7 in both groups, and 80% of microscopic PCF cases were caused by a herniated disc (herniated disc: 32, foraminal stenosis: 8) and UBE PCF were 75.7% (herniated disc: 25, foraminal stenosis: 8). The mean operative time in the microscopic PCF group (130.4 ± 38.5 min) was significantly longer than that in the UBE PCF group (105 ± 20.3 min) (*p* = 0.001). The mean length of hospital stay in the microscopic PCF group (8.0 ± 7.1 days) was also longer, although not statistically significant, than that in the UBE PCF group (5.6 ± 3.2 days) (*p* = 0.061). The mean estimated blood loss in the microscopic PCF group (141.3 ± 65.5 mL) was significantly greater than that in the UBE PCF group (39.5 ± 16.8 mL) (*p* = 0.001) (Table 1).

**Figure 6:**
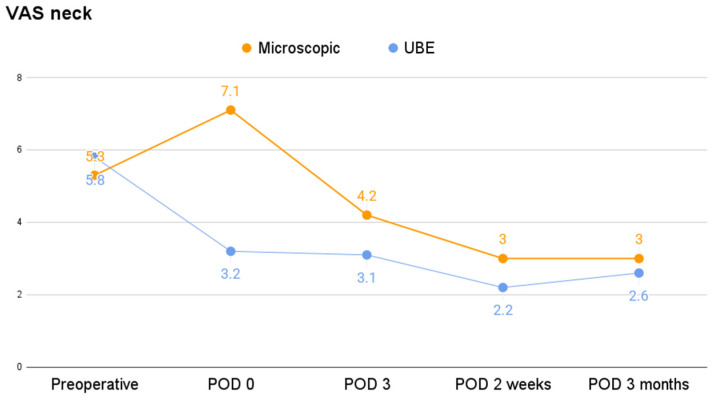
Comparison of postoperative Visual Analog Scale (VAS) scores for neck pain between the two groups.

**Figure 7:**
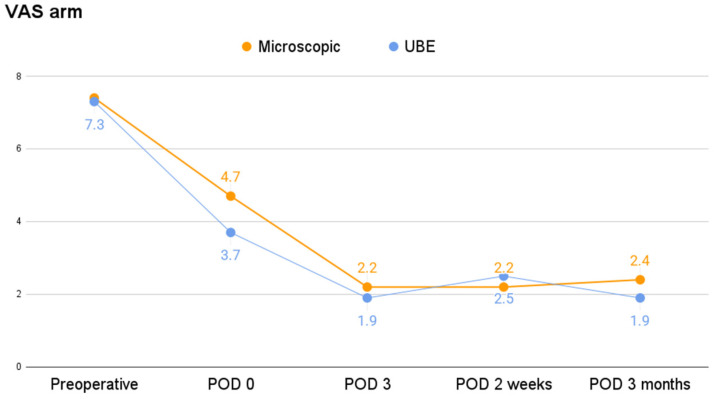
Comparison of postoperative Visual Analog Scale (VAS) scores for arm pain between the two groups.

The preoperative neck and arm VAS scores showed no significant differences between the two groups, indicating comparable baseline pain levels. However, postoperative neck VAS scores were significantly lower in the UBE PCF group than in the microscopic PCF group at POD 0, POD 3, and postoperative 2 weeks (*p* < 0.05). In addition, arm VAS was significantly lower in the UBE PCF group at POD 0 (*p* = 0.001), whereas no significant differences were observed at POD 3, postoperative 2 weeks, or postoperative 3 months (Figure 6 and Figure 7).

The NDI score also improved significantly more in the UBE PCF group than in the microscopic PCF group until postoperative 2 weeks, although the difference was no longer significant at postoperative 3 months. Because the preoperative NDI was significantly higher in the microscopic PCF group, additional change-score analyses were performed to account for baseline differences. The magnitude of NDI improvement, assessed by ΔNDI at postoperative 2 weeks and 3 months, did not differ significantly between the two groups (Table 2). While the overall trend of NDI improvement appeared similar between the two groups, the mean postoperative NDI values in the microscopic PCF group were influenced by several patients with unusually high scores, including one case with an NDI of 60. These outlier values likely elevated the group mean and may be associated with postoperative dysesthesia. A sensitivity analysis excluding the outlier case with an NDI score of 60 showed similar results, and the difference in NDI at postoperative 3 months remained statistically nonsignificant.

Recurrence, residual disc, revision surgery, and postoperative complications were analyzed as separate outcomes. Recurrence occurred in 1 patient (2.5%) in the microscopic PCF group and 1 patient (3.0%) in the UBE PCF group. Revision surgery was performed in 2 patients (5.0%) in the microscopic PCF group and 1 patient (3.0%) in the UBE PCF group. One case of residual disc requiring revision ACDF occurred in the UBE PCF group.

The overall complication rate was 12.5% in the microscopic PCF group and 6.1% in the UBE PCF group, with no statistically significant difference between the groups (*p* = 0.446). In the microscopic PCF group, postoperative complications occurred in five patients. These included two major complications, one postoperative hematoma and one ACDF revision performed four months later due to newly developed contralateral radiculopathy—and three minor complications, consisting of two cases of transient dysesthesia and one case of wound dehiscence.

In the UBE PCF group, complications occurred in two patients, including one major complication that required an ACDF revision because residual disc fragments could not be completely removed during the index procedure, resulting in persistent radicular symptoms (Table 3).

The change in cervical lordosis was not significantly different between the microscopic PCF (−0.29 ± 8.81) and UBE PCF (−1.66 ± 8.61) (*p* = 0.505). The change in segmental Cobb angle did not differ significantly between the microscopic PCF (0.12 ± 3.01) and UBE PCF (–0.92 ± 4.15) groups (*p* = 0.197). Likewise, the change in disc height was not significantly different between the microscopic PCF (0.29 ± 0.94) and UBE PCF (0.16 ± 0.83) groups (*p* = 0.134). In addition, no postoperative instability was observed in either group during follow-up (Table 4).

## 4. Discussion

Posterior cervical foraminotomy is highly effective in treating cervical radiculopathy and improving quality of life on the basis of previous reports [4,17]. Although, the purported drawback of muscle dissection and a partial facetectomy during the posterior cervical foraminotomy is the potential for postoperative neck pain which is thought to result from injury to the paraspinal muscles, cervical kyphosis and instability in patients with preexisting loss of cervical lordosis (Cobb angle < 10°) [15], and in particular there are times when two or three levels of posterior cervical foraminotomy are needed or considered. The UBE PCF achieves high-resolution visualization with only a small amount of muscle dissection and allows the use of almost all laminectomy instruments without restrictions. Previous studies have reported favourable clinical outcomes of minimally invasive endoscopic PCF, including UBE PCF, compared with other posterior cervical foraminotomy techniques [18,19,20]. Our study also has shown that postoperative neck pain and NDI were more improved in the UBE PCF group than in the microscopic PCF group until 2 weeks. Because the preoperative NDI was significantly higher in the microscopic PCF group, direct comparison of postoperative NDI values should be interpreted with caution. To reduce the influence of this baseline imbalance, we additionally performed change-score analyses using ΔNDI. The ΔNDI values at postoperative two weeks and three months did not differ significantly between the two groups, suggesting that the magnitude of functional improvement was comparable despite the difference in baseline NDI. Therefore, the apparent early difference in postoperative NDI should not be interpreted as definitive advantage of UBE PCF. It is important to note that the clinical superiority of UBE PCF observed in this study was mainly confined to the early postoperative period. Specifically, UBE PCF demonstrated advantages in postoperative neck pain and NDI improvement up to two weeks after surgery, whereas no significant differences were observed between the two groups at three months or later follow-up. Therefore, the clinical benefit of UBE PCF should be interpreted as an advantage in early recovery rather than as a long-term superiority over microscopic PCF. Accordingly, the findings of this study do not indicate overall clinical superiority of UBE PCF, but rather suggest that its main benefit may be limited to reduced early postoperative pain and faster short-term recovery.

In our study, postoperative neck pain was lower in the UBE PCF group during the early postoperative period. However, long-term clinical outcomes did not differ significantly between the two groups. In addition, operative time and estimated blood loss were lower, and hospital stay was numerically shorter in the UBE PCF group than in the microscopic PCF group. Although the overall complication rate was numerically lower in the UBE PCF group, this difference did not reach statistical significance. Therefore, the complication profile should be interpreted cautiously, and the present data do not demonstrate a clear superiority of UBE PCF in terms of complications. ACDF revision in the microscopic PCF group was performed 1 year and 4 months after surgery because of newly developed contralateral foraminal stenosis. In the UBE PCF group, ACDF revision was performed because of persistent radicular pain caused by residual disc fragments.

The two groups demonstrated no significant differences in sagittal alignment, segmental angle, or disc height change, and neither group showed radiographic evidence of instability. These radiologic parameters were evaluated to determine whether UBE PCF better preserved postoperative alignment and segmental stability compared with microscopic PCF. The anticipated advantage of maintaining sagittal alignment through posterior column preservation in the UBE group was not supported by the findings. This outcome is likely attributable to the inclusion of only single-level procedures in our cohort. Further long-term follow-up comparing radiological parameters is required to draw more definitive conclusions. In addition to conventional radiographic parameters, advanced image analysis may provide more objective and quantitative information for future studies of minimally invasive spine surgery. Radiomics is an imaging-based analytic method that extracts quantitative features from medical images and may help characterize tissue status, disease heterogeneity, and treatment response beyond visual assessment alone. Similarly, artificial intelligence-based image analysis and molecular imaging techniques, including PET or SPECT, may contribute to improved diagnosis, patient selection, and treatment planning in selected inflammatory or degenerative spinal conditions. Although these approaches were beyond the scope of the present study, their integration with clinical and radiologic outcomes may support more individualized decision-making and precision medicine in future investigations of cervical foraminal disc herniation or stenosis [21,22,23]. Recent studies on fully endoscopic and other minimally invasive spinal procedures also support the broader trend toward reduced tissue disruption and faster postoperative recovery.

Although no dural tear occurred in the present study, this complication remains an important technical consideration in UBE procedures. Although endoscopic surgery requires technical proficiency and is generally associated with a longer learning curve than microscopic surgery, UBE has a relatively shorter learning curve compared with full-endoscopic techniques. Because UBE is a water-based procedure, however, a dural tear can result in increased intracranial pressure, potentially leading to serious complications. This risk is particularly relevant in cervical procedures, given the anatomical proximity to the cranial cavity, where intracranial pressure can rise more rapidly following a dural breach. Therefore, if a dural tear occurs, the procedure should be halted immediately and appropriate dural repair should be performed.

When these considerations are properly addressed, UBE foraminotomy may serve as a viable alternative to microscopic foraminotomy.

Because the two procedures were performed during different time periods rather than concurrently, the observed differences may have been affected not only by the surgical technique itself but also by temporal factors such as accumulated surgical experience and changes in perioperative management.

## 5. Limitations

First, this study has an inherent limitation due to its retrospective design and relatively short follow-up duration. Second, although the actual follow-up periods were similar between the two groups, the procedures were performed during different time frames. Microscopic PCF was mainly performed in the earlier period, whereas UBE PCF was introduced and performed in the later period. Therefore, temporal bias and learning-curve bias may have influenced the results. Improvements in surgical experience, patient selection, perioperative management, and institutional practice patterns over time may have contributed to the observed early postoperative advantages in the UBE PCF group. In addition, because UBE PCF was a newer technique during the study period, surgeon proficiency and case selection may have affected the comparative outcomes. This limitation should be carefully considered when interpreting the apparent advantages of UBE PCF, and the results should not be interpreted as definitive evidence of superiority. Third, the study included a relatively small number of patients and was conducted at a single centre, which may limit the generalizability of the findings. Therefore, further prospective randomized controlled trials are warranted to validate these results. Fourth, formal interobserver and intraobserver reliability analyses, such as ICC, were not performed for the radiologic measurements. Although these parameters have been reported to show acceptable reproducibility in previous studies, this may have influenced the precision of the radiologic outcome assessment. Fifth, repeated-measures analysis such as repeated-measures ANOVA or a linear mixed-effects model was not performed; therefore, the longitudinal changes in VAS and NDI should be interpreted cautiously.

## 6. Conclusions

Both microscopic and UBE posterior cervical foraminotomy are effective and safe procedures for the treatment of cervical foraminal disc herniation and stenosis. In this study, UBE PCF was associated with lower early postoperative neck pain, shorter operative time, numerically shorter hospital stays, and less estimated blood loss. However, long-term clinical and radiologic outcomes were comparable between the two groups. Therefore, UBE PCF should be considered a safe and effective minimally invasive alternative to microscopic PCF, particularly for facilitating early postoperative recovery, rather than a definitively superior procedure.

## Figures and Tables

**Table 1:** Demographics and characteristics of the operations.

	Microscopic PCF (*N* = 40)	UBE PCF (*N* = 33)	*p*-Value
Age (yr, mean ± SD)	56.6 ± 9.7 (39–78)	56.1 ± 10.0 (41–75)	0.855
Sex (*n*, %)			0.711
Male	25	22	
Female	15	11	
Pathology (*n*, %)			0.663
Disc herniation	32	25	
Foraminal stenosis	8	8	
Operation level (*n*, %)			0.544
C3/4	3 (7.5%)	0 (0%)	
C4/5	1 (2.5%)	1 (3%)	
C5/6	9 (22.5%)	10 (30.3%)	
C6/7	21 (52.5%)	16 (48.5%)	
C7/T1	6 (15%)	6 (18.2%)	
Operation time (min, mean ± SD)	130.4 ± 38.5	105.0 ± 20.3	0.001
Length of hospital stay (days, mean ± SD)	8.0 ± 7.1 *	5.6 ± 3.2	0.061
Estimated blood loss (mL, mean ± SD)	141.3 ± 65.5	39.5 ± 16.8	0.001
Average Follow up period (m, mean ± SD)	33.0 ± 7.6	29.9 ± 5.9	0.061

PCF = posterior cervical foraminotomy, SD = standard deviation, UBE = Unilateral Biportal Endoscopy. * The mean hospital stay was affected by one outlier case with 49 hospital days.

**Table 2:** Clinical outcomes and change-score analyses following Microscopic PCF and UBE PCF.

	Microscopic PCF (*N* = 40)	UBE PCF (*N* = 33)	*p*-Value ^†^
NDI			
Preoperative	31.2 ± 8.0	27.5 ± 5.0	0.029
POD 2 weeks	14.1 ± 8.6	8.9 ± 6.1	0.002
POD 3 months	6.9 ± 11.1 ^‡^	3.6 ± 10.4	0.150
ΔNDI (Preop to POD 2 weeks)	−17.1 ± 8.3	−18.6 ± 7.5	0.412
ΔNDI (Preop to POD 3 months)	−24.3 ± 10.2	−23.9 ± 9.8	0.781
VAS neck			
Preoperative	5.3 ± 1.4	5.8 ± 1.4	0.139
POD 0	7.1 ± 0.9	3.2 ± 1.2	<0.001
POD 3	4.2 ± 1.0	3.1 ± 0.4	<0.001
POD 2 weeks	3.0 ± 0.9	2.2 ± 1.1	0.002
POD 3 months	3.0 ± 1.8	2.6 ± 1.1	0.268
VAS arm			
Preoperative	7.4 ± 1.4	7.3 ± 1.1	0.790
POD 0	4.7 ± 1.3	3.7 ± 1.2	0.001
POD 3	2.2 ± 1.1	1.9 ± 0.7	0.192
POD 2 weeks	2.2 ± 1.2	2.5 ± 1.0	0.279
POD 3 months	2.4 ± 1.8	1.9 ± 0.7	0.077

PCF = posterior cervical foraminotomy, VAS = Visual Analog Scale, POD = postoperative day, NDI = neck disability index. ^†^ Statistical significance was determined using independent *t*-test between the two groups. ^‡^ Mean NDI value in the microscopic group was influenced by a few outliers with high scores (including one case with NDI 60), possibly due to postoperative dysesthesia.

**Table 3:** Postoperative complications.

	Microscopic PCF (*N* = 40)	UBE PCF (*N* = 33)	*p*-Value
Total	5 (12.5%)	2 (6.1%)	0.446
Major complication			
Postoperative hematoma	1 (2.5%)	0 (0.0%)	
ACDF revision	1 (2.5%)	1 (3.0%)	
Minor complication			
Transient dysesthesia	2 (5.0%)	1 (3.0%)	
Wound dehiscence	1 (2.5%)	0 (0.0%)	

PCF = posterior cervical foraminotomy, ACDF = anterior cervical discectomy and fusion.

**Table 4:** Radiological outcomes following microscopic PCF and UBE PCF.

	Microscopic PCF (*N* = 40)	UBE PCF (*N* = 33)	*p*-Value
Δ Cervical lordosis (°, mean ± SD)	−0.29 ± 8.81	−1.66 ±8.61	0.505
Δ Segmental Cobb angle (°, mean ± SD)	0.12 ± 3.01	−0.92 ± 4.15	0.197
Δ Disc height (mm, mean ± SD)	0.29 ± 0.94	0.16 ± 0.83	0.134
Postoperative instability (*n*, %)			
Present	0 (0%)	0 (0%)	
None	40 (100%)	33 (100%)	

Minus degree indicates kyphosis. Δ = postoperative value − preoperative value, PCF = posterior cervical foraminotomy, ACDF = anterior cervical discectomy and fusion.

## Data Availability

The data presented in this study are available from the corresponding author upon reasonable request. The data are not publicly available due to privacy and ethical restrictions.
